# Role of the oral-gut microbiota axis in pancreatic cancer: a new perspective on tumor pathophysiology, diagnosis, and treatment

**DOI:** 10.1186/s10020-025-01166-w

**Published:** 2025-03-18

**Authors:** Xuanchi Guo, Yuhan Shao

**Affiliations:** 1 School of Stomatology, Shandong University, No. 44-1 Wenhua West Road, Jinan City, Shandong Province China; 2 State Key Laboratory of Oral Diseases & National Center for Stomatology & National Clinical Research Center for Oral Diseases, West China Hospital of Stomatology, Sichuan University, Chengdu, 610041 China

**Keywords:** Oral-gut microbiota axis, Pancreatic cancer, Immune regulation, Early diagnosis, Theraputic application

## Abstract

Pancreatic cancer, one of the most lethal malignancies, remains challenging due to late diagnosis, aggressive progression, and therapeutic resistance. Recent advances have revealed the presence of intratumoral microbiota, predominantly originating from the oral and gut microbiomes, which play pivotal roles in pancreatic cancer pathogenesis. The dynamic interplay between oral and gut microbial communities, termed the “oral-gut microbiota axis,” contributes multifacetedly to pancreatic ductal adenocarcinoma (PDAC). Microbial translocation via anatomical or circulatory routes establishes tumor-resident microbiota, driving oncogenesis through metabolic reprogramming, immune regulation, inhibition of apoptosis, chronic inflammation, and dysregulation of the cell cycle. Additionally, intratumoral microbiota promote chemoresistance and immune evasion, further complicating treatment outcomes. Emerging evidence highlights microbial signatures in saliva and fecal samples as promising non-invasive diagnostic biomarkers, while microbial diversity correlates with prognosis. Therapeutic strategies targeting this axis—such as antibiotics, probiotics, and engineered bacteria—demonstrate potential to enhance treatment efficacy. By integrating mechanisms of microbial influence on tumor biology, drug resistance, and therapeutic applications, the oral-gut microbiota axis emerges as a critical regulator of PDAC, offering novel perspectives for early detection, prognostic assessment, and microbiome-based therapeutic interventions.

## Introduction

Pancreatic cancer, the seventh leading cause of cancer-related deaths globally, is predominantly (approximately 90%) pancreatic ductal adenocarcinoma (PDAC) (Stoffel et al. 2023). Its incidence and mortality rates have risen steadily over the past 25 years, with an average annual increase of 1.1%, posing a growing public health burden worldwide (Ramai et al. 2024). Moreover, The disease is often asymptomatic in its early stages, leading to late diagnosis and poor prognosis, with a 5-year survival rate below 10% and significant regional disparities (Hu et al. 2021). Addressing this highly fatal disease requires innovative approaches to early screening and treatment. Recent advances in PDAC research, particularly through next-generation sequencing, have revealed the presence of intratumoral microbiota in tissues once considered sterile. These microorganisms, known as intratumoral microbiota are found to be critical in tumor development and progression.

The origin of intratumoral microbiota in PDAC remains debated, but the oral cavity and gut are widely recognized as the most abundant sources (Guan et al. 2023). The oral microbiota, shaped by factors such as nutrients, oxygen, and temperature, evolves from birth to form a stable community dominated by *Streptococcus*, *Neisseria*, *Corynebacterium*, *Rothia*, *Actinomyces*, *Porphyromonas*, and *Fusobacterium* (Zaura et al. 2009; Khor et al. 2021). Similarly, the gut microbiota, after a series of developments, forms a healthy microbial community primarily composed of *Firmicutes* (30–50%), *Bacteroidetes* (20–40%), *Actinobacteria* (1–10%), and *Proteobacteria* (Gagnière et al. 2016), some of which have essential functions like nutrient conversion, vitamin production, immune tolerance, and protection against neurological diseases (Weiss and Hennet 2017; Giuffrè et al. 2020). However, changes in environmental factors, such as nutrition, the abuse of antibiotic, radiation, or chemotherapy, can disrupt this balance, potentially contributing to cancer development. Due to anatomical features, although not identical, the oral and gut microbiomes are interconnected. Oral bacteria can translocate to the gut via circulation or the digestive tract, while gut bacteria may migrate to the oral cavity. This bidirectional exchange is implicated in gastrointestinal diseases, with oral pathogens such as *Staphylococcus*, *Porphyromonas*, *Fusobacterium*, *Actinomyces*, and *Neisseria* frequently detected in the guts of affected patients (Khor et al. 2021; Kitamoto et al. 2020).

These findings suggest that the oral and gut microbiomes are closely intertwined and mutually influential. We therefore aim to explore the potential role of the “oral-gut microbiota axis” in PDAC, offering a new perspective on the pathophysiology, diagnosis, prognosis, and treatment of the disease.

## The relationship between the oral-gut microbiota axis and the origin of intratumoral microbiota in PDAC

The origin of intratumoral microbiota in PDAC remains controversial, with the gut and oral cavity being the most widely accepted sources. Anatomically, the pancreatic duct exits the right side of the pancreas, traverses the posterior medial wall of the descending duodenum, and merges with the common bile duct, opening at the major duodenal papilla. This structure facilitates the retrograde entry of gut microbiota into the pancreas(Table 1). Early evidence from Nielsen et al. (2006) detected *Helicobacter pylori* DNA in 75% of PDAC patients’ tumor tissues (Nilsson et al. 2006). Okuda et al. also found a strong co-localization of representative bacteria from pancreatic juice in PDAC tissues (Okuda et al. 2022). Additionally, experimental studies in PDAC mice models have shown that fungal gavage results in detectable fungi in tumor tissues through staining or immunological methods (Alam et al. 2022; Aykut et al. 2019). However, comparative analyses of microbiota from tumor tissues, duodenal fluid, and duodenal tissues in post-surgical PDAC patients revealed that while some similarities exist between duodenal and PDAC microbiomes, certain microbiota in PDAC tissues were absent in the duodenum (Thomas and Jobin 2020), shifting research focus toward the oral cavity.

Oral microbiota typically spread to the gastrointestinal tract and can retrograde into the pancreas through the pancreatic duct, with some microbes like *Fusobacterium nucleatum* potentially entering via blood or lymphatic drainage (Parhi et al. 2020; Widdison et al. 1994; Tan et al. 2022). Previous studies have shown that at the phylum level, both intratumoral and oral microbiota in PDAC patients are dominated by *Firmicutes*, *Proteobacteria*, and *Bacteroidetes*. Among them, *Porphyromonas gingivalis* has been most extensively studied. Multiple findings suggest that *P. gingivalis* is present in the PDAC microenvironment (Meier et al. 2019; Farrell et al. 2012), and its presence in the pancreas has been confirmed through flow cytometry and fluorescence in situ hybridization (FISH). Interestingly, while *Proteobacteria* are highly abundant in PDAC tissues, they are not particularly enriched in the oral cavity but are relatively common in the gut. These studies indicate that intratumoral microbiota in PDAC are closely intertwined with the oral and gut microbiota, supporting the hypothesis that the oral-gut microbiota axis could serve as the source of these intratumoral microorganisms.

Comparative studies using HOMIM technology have revealed significant differences in oral microbiota between PDAC patients (*n* = 10) and healthy controls (*n* = 10), with 31 bacterial species increased and 25 decreased in the saliva of patients. The *Firmicutes* phylum exhibited the most varied changes, including 34 different genera/clusters, with *Streptococcus* being the most diverse genus, containing 13 different species/groups (Farrell et al. 2012). As awareness of periodontitis has grown, the positive correlation between periodontitis and PDAC has also been increasingly recognized (Michaud 2013; Ahn et al. 2012). *Porphyromonas gingivalis*, which plays a significant role in periodontitis, has attracted research attention. Ahn et al. demonstrated that elevated antibody levels against P. gingivalis were associated with a threefold increased risk of death from oral and gastrointestinal cancers using NHANES III data (Michaud et al. 2013). In a large prospective cohort study with up to 9 years of follow-up, a nested case-control study on 405 PDAC patients and 410 controls matched by age, time of blood collection, health status, gender, and study center revealed that individuals with higher *P. gingivalis* antibody levels had twice the risk of developing PDAC compared to the control group (OR = 2.38, 95% CI = 1.16–4.90) (Kohi et al. 2022).Similarly, the gut microbiota in PDAC patients also undergoes significant changes. Following the discovery of the link between *Helicobacter pylori* and gastric cancer, Raderer et al. found a correlation between *H. pylori* and the risk of PDAC. Furthermore, comparative analysis of 134 normal pancreatic controls, 98 pancreatic cyst patients, and 74 PDAC patients showed increased abundance of *Bifidobacterium*, *Roche*, and *Fusobacterium* in PDAC patients (Thomas et al. 2018). Additional studies have reported elevated levels of *Acinetobacter*, *Pseudomonas*, *Sphingomonas*, and *Citrobacter freundii* in PDAC patients, with microbial genetic analysis suggesting their potential role in tumor-related inflammation and tumorigenesis. Notably, Jaideep et al. identified *Proteobacteria*, particularly *β-Proteobacteria* and *γ-Proteobacteria*, as significant microorganisms associated with PDAC, showing positive correlation with metastasis (Chakladar et al. 2020; Guo et al. 2021; Sepich-Poore et al. 2022).

**Table 1:** The characterization of the intratumoral microbiota in PC

Research groups	Methods	Number of samples	Associated microbiota (same)	Associated microbiota (different)
Nilsson et al. 2006	PCR	40 exocrine PC, 14 neuroendocrine cancer 8 multiple endocrine neoplasia, 5 chronic pancreatitis, 10 benign pancreatic disease and 7 normal pancreas samples	–	*Helicobacter pylori*
Aykut et al. 2019	18 S ITS sequencing	The gut (*n* = 14 biologically independent samples) and intrapancreatic (*n* = 11 biologically independent samples) mycobiomes in 30 week-old KC mice	*Candida*, *Saccharomyces*, or *Aspergillus*	*Malassezia*
Kohi et al. 2022	16 S rRNA sequencing	134 normal pancreas control individual, 98 pancreatic cyst patient, and 74 PDAC patient samples	–	*Enterococcaceae*, *LactobacillaceaeBifidobacteriaceae*, *Nakaseomyces* and *Skeletocutis*
Farrell et al. 2012	Human Oral Microbe Identification Microarray	10 resectable patients with pancreatic cancer and 10 matched healthy controls	–	*Neisseria elongata*, *Streptococcus mitis*, *Granulicatella adiacens*, *Phylum Firmicutes*, *Streptococcus salivarius*
Michaud et al. 2013	Immunoblot array	405 pancreatic cancer cases and 416 matched controls	–	*Porphyromonas gingivalis*
Guo et al. 2021	16 S rRNA sequencing, metagenomic sequencing and PCR	17 basal-like, 23 hybrid and 22 classical pancreatic tumors	–	*Acinetobacter*, *Pseudomonas* and *Sphingopyxis*

## The oral-gut microbiota axis promotes the occurrence and development of PDAC

The oral-gut microbiota axis represents a complex ecosystem of microorganisms that colonize both the oral cavity and gastrointestinal tract, forming a dynamic symbiotic relationship with the human host. This microbial community, comprising approximately ten times more cells than human somatic cells and encoding over a million genes, has been increasingly recognized as a crucial regulator in human physiology and pathology. Particularly in PDAC, the oral-gut microbiota axis exerts its influence through multifaceted mechanisms, with immune regulation emerging as an significant player in tumor development and progression. Beyond immunological mechanisms, the oral-gut microbiota axis contributes to PDAC pathogenesis through metabolic reprogramming, induction of chronic inflammation, regulation of cellular apoptosis, and perturbation of cell cycle control. The profound impact of this microbial axis on PDAC biology has led to its recognition as a potential “second genome” of cancer (Hermann et al. 2002), mirroring the conceptual framework established for the human microbiome. Understanding these complex mechanisms, holds significant promise for advancing targeted therapies and overcoming therapeutic resistance, potentially revolutionizing the management of PDAC through microbiome-based interventions and personalized treatment approaches.

### The impact of metabolites

Metabolite research remains a pivotal aspect of microbiota studies. Despite the relatively small biomass of intratumoral microbiota, their metabolites play significant roles in PDAC and other tumors. Lipopolysaccharides (LPS), essential components of Gram-negative bacterial cell walls, promote tumor progression through TLR binding and subsequent activation of downstream signaling molecules, mediating inflammation and immune suppression. Hermann et al. discovered that LTS, another Gram-negative bacterial component, binds to CD14 and triggers excessive inflammatory cytokine secretion (Wei et al. 2019). Deoxycholic acid (DCA), produced by gut bacteria through 7α-dehydroxylation of secondary bile acids, induces PDAC by activating EGFR, MAPK, and STAT3 signaling pathways, while also causing DNA damage (Sugimoto et al. 2022). Furthermore, nitrosamine compounds produced by *Candida albicans* may contribute to carcinogenesis through prolonged exposure.

Recent studies have demonstrated reduced levels of short-chain fatty acids (SCFAs) and gut peptides in PDAC patients. The depletion of SCFAs, particularly propionate, compromises mucosal barrier integrity and reduces mucosa-associated invariant T cells (MAITs), which Chie Su et al. found to exhibit potential anti-tumor properties (Pierre et al. 2023). Notably, gut peptides, beyond their traditional roles in appetite and digestion, have shown antifungal activity. Specifically, PYY, a peptide expressed by Paneth cells, demonstrates significant antifungal effects against *Candida albicans* (Aykut et al. 2019), though its activity against PDAC-associated *Malassezia* requires further investigation.

### Immune regulation

**Fig. 1:**
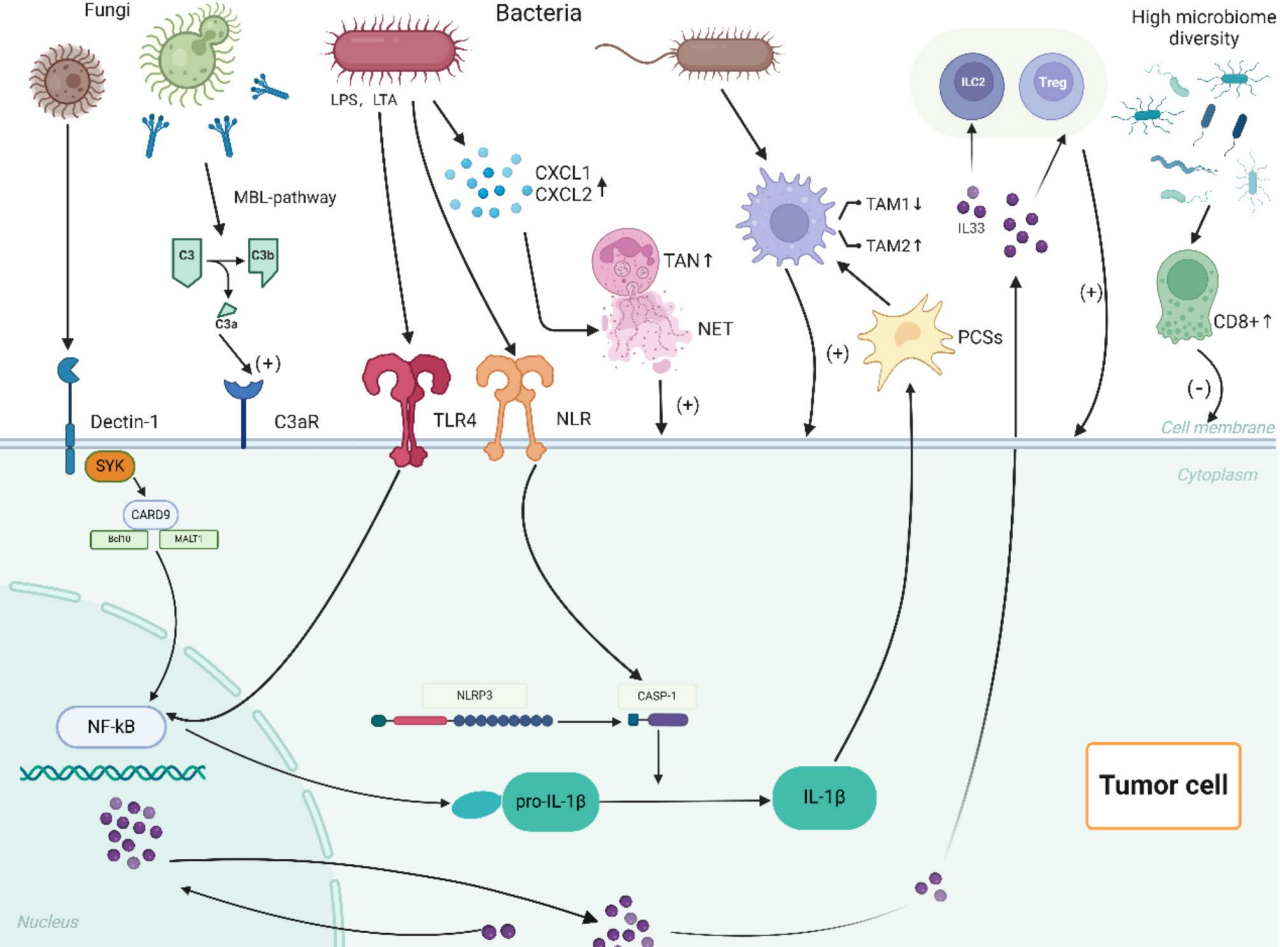
The intratumour microbiome-immune-PDAC axis. Intratumoral fungi (such as *Malassezia* or *Chaetomium*) and their acellular extracts promote the secretion of interleukin (IL)-33 through the dectin-1 receptor-mediated Src-Syk-CARD 9 pathway. The secretion of IL-33 enriches helper T cells 2, group 2 innate lymphoid cells, and T lymphocytes in the tumor microenvironment (TME), thereby promoting the progression of pancreatic ductal adenocarcinoma (PDAC). Additionally, intratumoral fungi can activate the complement cascade of complement 3 (C3) via the “lectin pathway,” leading to the breakdown of C3 into C3a and C3b. C3a promotes the proliferation of PDAC cells by binding to C3a receptors on cancer cell surfaces. Intratumoral bacteria inhibit TAM 1 polarization by activating toll-like receptors (TLRs) on cell surfaces, accompanied by an increase in TAM 2 transformation, ultimately facilitating tumor cell proliferation and tumor angiogenesis. They also promote the secretion of IL-1β through TLR 4 and NLR on PDAC cell surfaces via NF-κB and CASP-1, respectively. The secretion of IL-1β indirectly promotes TAM 2 activation by activating pancreatic stellate cells. Intratumoral bacteria also promote the secretion of neutrophil chemokines in the TME of PDAC, leading to the enrichment of tumor-associated neutrophils 2 (TAN 2) in the TME. Finally, the high diversity of the intratumoral microbiome enhances the activity of CD 8 ^+^ T cells to suppress PDAC

The innate and adaptive immune systems are vital components of the body’s defense mechanisms, and both are involved in tumor surveillance, which monitors and eliminates abnormal cells. Over time, the microbiota in the oral-gut axis has established a delicate balance with the immune system. However, dysbiosis in cancer patients disrupts this balance, and microbes, along with their metabolites, influence the occurrence of pancreatic ductal adenocarcinoma (PDAC) by regulating innate and adaptive immunity through the intratumoral microbiota-immune-PDAC axis(Fig. 1).

#### Innate immune system

The innate immune system is the body’s first line of defense, consisting of physiological barriers, innate immune cells (such as macrophages, neutrophils, and NK cells), and innate immune molecules (such as complement proteins, mannose-binding lectin (MBL), and interferon-gamma). Innate immune cells and molecules are the primary targets for microbial interactions.

##### The complement system

The complement system is a critical part of the innate immune response. Its primary function is to recognize and clear pathogens, dead cells, and cell debris. Some complement proteins remain in an activated state for extended periods, providing long-term surveillance of abnormal cells and participating in “immune surveillance.” The complement system is regulated by three pathways: the classical pathway, the alternative pathway, and the lectin pathway. Regardless of the pathway, the end result is the formation of C3 convertase, which cleaves C3 into C3a and C3b. Numerous studies have shown that C3a plays a pro-tumorigenic role. Thus, when the complement system becomes dysregulated, excessive production of C3a can promote tumor formation. Aykut et al. found that in KPC mice, intratumoral fungi, particularly *Malassezia*, activated the complement cascade through the lectin pathway (Suzuki et al. 2022). Fungal cell wall glycans bind to MBL, and with the participation of MBL-associated serine proteases 1 and 2 (MASP-1 and MASP-2), C3 convertase is formed. C3 convertase then cleaves C3 into C3a, which binds to the C3aR receptor on the surface of PDAC cells, directly promoting cell proliferation and activating the ERK pathway to enhance epithelial-mesenchymal transition (EMT) (Gadwa et al. 2021). Additionally, in some in vitro experiments, C3aR was found to be expressed on CD4 + T cells, potentially playing a role in the stability of CD4 + T cells and the regulation of Foxp3 + T regulatory cells (Pushalkar et al. 2018).

##### PRRs

Pattern recognition receptors (PRRs) are a class of receptors primarily expressed on the surface of innate immune cells. They are non-clonally distributed and can recognize one or more pathogen-associated molecular patterns (PAMPs) or damage-associated molecular patterns (DAMPs). The recognition and binding between PRRs and PAMPs or DAMPs is key to initiating the innate immune response. Among the PRRs related to tumors, Toll-like receptors (TLRs) are particularly representative. TLRs (especially TLR2, TLR3, TLR4, TLR7, and TLR9) can recognize PAMPs such as LPS, LTA, and flagellin, activating NF-κB, Notch, MAPK, and STAT3 signaling pathways, leading to the recruitment and infiltration of inflammatory cytokines, and even promoting tumor development (Knight et al. 2023). This has proven correct by experiments blocking TLRs and is gradually being applied in clinical trials (Li et al. 2020). Similarly, another class of PRRs, nucleotide-binding oligomerization domain-like receptors (NLRs), has also garnered research interest. These receptors (such as NLRP1, NLRP3, and NLRP4) recognize microbial molecules, activate the caspase-1 inflammasome, and subsequently release interleukin (IL)-1β and IL-18 (Bai et al. 2023). Furthermore, NLRs promote the formation of autophagosomes via NF-κB, P38 MAPK, and interferon signaling pathway, and autophagosomes have been recently found to be associated with the activation of pancreatic stellate cells, a major cell type in PDAC (Demkow 2021).

##### Innate immune cells

Neutrophils are one of the primary members of innate immune cells. They can phagocytize and kill bacteria, as well as participate in inflammatory responses. Generally, neutrophils exert their functions through three main mechanisms: phagocytosis, degranulation, and the release of neutrophil extracellular traps (NETs). NETs are extracellular DNA structures released by neutrophils that are decorated with antimicrobial enzymes to capture and kill pathogens. However, in tumors, NETs can play a harmful role. Evidence shows that NETs can awaken dormant cancer cells, promote uncontrolled proliferation, and eventually lead to tumor recurrence. Similarly, a report indicated that NETs could enhance the invasiveness of tumor cells by releasing matrix metalloproteinases (MMPs) and inducing the release of high-mobility group box 1 (HMGB1), promoting epithelial-mesenchymal transition (EMT) (Tan et al. 2022). In PDAC, *P. gingivalis*, an important pathogenic bacterium in the oral cavity, can promote the secretion of chemokines (CXCL1 and CXCL2) in the tumor microenvironment (TME), thereby recruiting tumor-associated neutrophils (TANs) in the TME to release NETs (Wu et al. 2024). However, some studies have also confirmed that neutrophils and their NET can kill tumor cells to some extent. Therefore, whether neutrophils have a cancer promoting or cancer suppressing role in the tumor microenvironment is still a hot topic of debate among researchers. Rencently, a new breakthrough has emerged: a study published in *Cell* by Wu et al. used single-cell transcriptomics of neutrophils from 225 samples across 17 cancers (including liver cancer, cholangiocarcinoma, and gallbladder cancer) from 143 patients. The study established a comprehensive map of tumor-associated neutrophils (TANs), revealing their surprising heterogeneity, diversity, and plasticity (Ge and Wu 2023). Notably, HLA-DR + CD74 + neutrophils were positively correlated with better prognosis in several cancers, providing new strategies for sensitizing tumors to immunotherapy.

Another branch of innate immune cells, macrophages, also plays a crucial role in the tumor microenvironment (TME). Chemokines, cytokines, and exosomes in the TME induce and recruit macrophages. When large numbers of activated macrophages infiltrate the tumor stroma, they are termed as tumor-associated macrophages (TAMs). Like normal macrophages, TAMs can also be polarized into two phenotypes: M1 classically activated macrophages (M1) or M2 alternatively activated macrophages (M2). Numerous studies have shown that M1 macrophages can express TNF-α, IL-1β, IL-6, IL-12, and IL-23, directly acting on tumor cells and promoting a Th1 response to inhibit tumors (Hermann et al. 2002). Additionally, M1 macrophages can upregulate genes related to antigen processing and presentation, increase co-stimulatory molecules and major histocompatibility complex II (MHC-II) expression, thereby enhancing T-cell-mediated tumor cell killing. In contrast, M2 macrophages promote a Th2 response, secreting IL-4, IL-13, IL-10, IL-8, TGF-β, as well as growth factors like epidermal growth factor (EGF), platelet-derived growth factor (PDGF) and basic fibroblast growth factor (BFGF). These factors not only stimulate tumor cell proliferation but are also involved in tumor angiogenesis. Furthermore, the secretion of arginase-1 by TAM2 reduces L-arginine levels, which are crucial for the proliferation of T cells and NK cells, while also recruiting Foxp3 + regulatory T cells (Tregs), thus contributing to tumor progression (Alam et al. 2022; Qiao and Fu 2022). Unfortunately, dysbiosis in the TME can promote the polarization of macrophages toward the M2 phenotype. For example, *Bifidobacterium pseudolongum* promotes the polarization of TAMs toward M2 by acting on TLR2 and ILR5, while reducing TAM1, ultimately promoting tumor growth (Guan et al. 2023). Additionally, Ge et al. have found that the hypoxic environment caused by microbial dysbiosis also promotes M2 polarization (Alam et al. 2022).

In addition, regarding type II innate lymphoid cells (ILC2s), fungi (*Malassezia* or *Alternaria*) and their acellular extracts in PDAC can promote IL-33 secretion via the dectin-1 receptor-mediated Src-Syk-CARD9 pathway, leading to the infiltration of ILC2s (Moral et al. 2020). Most studies suggest that ILC2s are pro-tumor cells, promoting tumor growth through IL-5. However, one experiment proposed that the ILC2-CD103 + dendritic cell-CD8 + T cell axis can inhibit PDAC progression (Tan et al. 2022). We therefore hypothesize this dual role of ILC2 cells in PDAC may provide clues for the application of the promoting/inhibitory effects of the intratumoral microbiome under different immunogens to the anti-tumor effects of intratumoral fungi.

#### Adaptive immune system

CD8 + T cells are the main effector cells of the adaptive immune system. They kill target cells by recognizing tumor antigens presented by MHC-I molecules. In mouse experiments, researchers gavaged PDAC mice with *Alternaria* and *P. gingivalis*, finding a significant reduction in CD8 + T cells within the TME (Alam et al. 2022; Riquelme et al. 2019). Antibiotic ablation experiments also confirmed that reducing microbiota increased the CD8 + T cell ratio and the number of cytotoxic CD8 + T cells. Similarly, researchers reported that in antibiotic-ablated mice, both CD4 + and CD8 + T cells in the TME exhibited increased expression of surface molecules PD-1 and CD44. Further studies collected T cells from the tumors of both control and antibiotic-treated KPC mice (a pancreatic cancer model carrying mutations in the Kras and Trp53 genes) and transferred these T cells into a cohort of KPC mice. The results showed that tumor-infiltrating T cells from control mice did not confer protective effects, whereas T cells from antibiotic-treated mice reduced the tumor burden by approximately 50%. This suggests that microbiota may influence adaptive immunity by inducing T-cell exhaustion (Knight et al. 2023). Additionally, the activation of T cells is linked to antigen-presenting cells (APCs) such as macrophages, but microbiome-entrained macrophages show a significantly reduced capacity for antigen presentation, weakening T-cell activation. Interestingly, multiple studies on PDAC prognosis have shown that patients with higher intratumoral microbial α-diversity have higher CD3 + and CD8 + T cell densities within the TME, longer lifespans, and better prognoses. These findings suggest that intratumoral microbiota might not only promote tumors but may also inhibit them, raising the possibility that identifying these “good” microbes could open a new avenue for adjunctive therapy in PDAC (Kostic et al. 2013).

Comprehensively, it is important to note that a single microbe may influence both innate and adaptive immunity through multiple pathways. For instance, *F. nucleatum* selectively recruits dendritic cells (especially CD103 + regulatory DCs), TAMs, MDSCs, and CD11b myeloid cells to alter the tumor immune microenvironment and promote immune evasion. *F. nucleatum* also selectively induces the expansion of MDSCs (e.g., M-MDSCs and G-MDSCs), which in turn significantly inhibit CD4 + T cell activity by secreting arginase-1 and iNOS (Wang and Fang 2023). Additionally, *F. nucleatum*’s FapA protein binds to the TIGIT receptor, directly suppressing the cytotoxicity of T cells and NK cells, thereby disrupting the anti-tumor response (Gnanasekaran et al. 2020).

Similarly, a single pathway may be regulated by multiple microbes. For instance, the TLR pathway has been mentioned in studies involving *Porphyromonas gingivalis*, *Fusobacterium nucleatum*, *Bifidobacterium pseudolongum*, and *Megasphaera* (Wang and Fang 2023; Huang et al. 2022; Kashyap et al. 2021). Generally speaking, complex interactions exist within the intratumoral microbiome-immune-PDAC axis, and further analysis and interpretation of these intricate relationships will be of great significance for understanding the role of intratumoral microbiota in PDAC.

### Apoptosis

Apoptosis is a type of programmed cell death, regulated by genes, and plays a crucial role not only in the growth and development of organisms and the homeostasis of tissues and organs but also in various pathological processes. Cell apoptosis is one of the intrinsic surveillance mechanisms of cells, capable of promptly eliminating non-functional, harmful, and abnormal cells. Apoptosis has long been considered an important mechanism for preventing the emergence of tumors. Conversely, one of the characteristics of tumor cells is the inhibition of apoptosis. In recent years, various experiments have revealed that certain intra-tumoral microorganisms from the oral-gut microbiome axis actually play a facilitator role in the anti-apoptotic process of tumor cells.

**Fig. 2:**
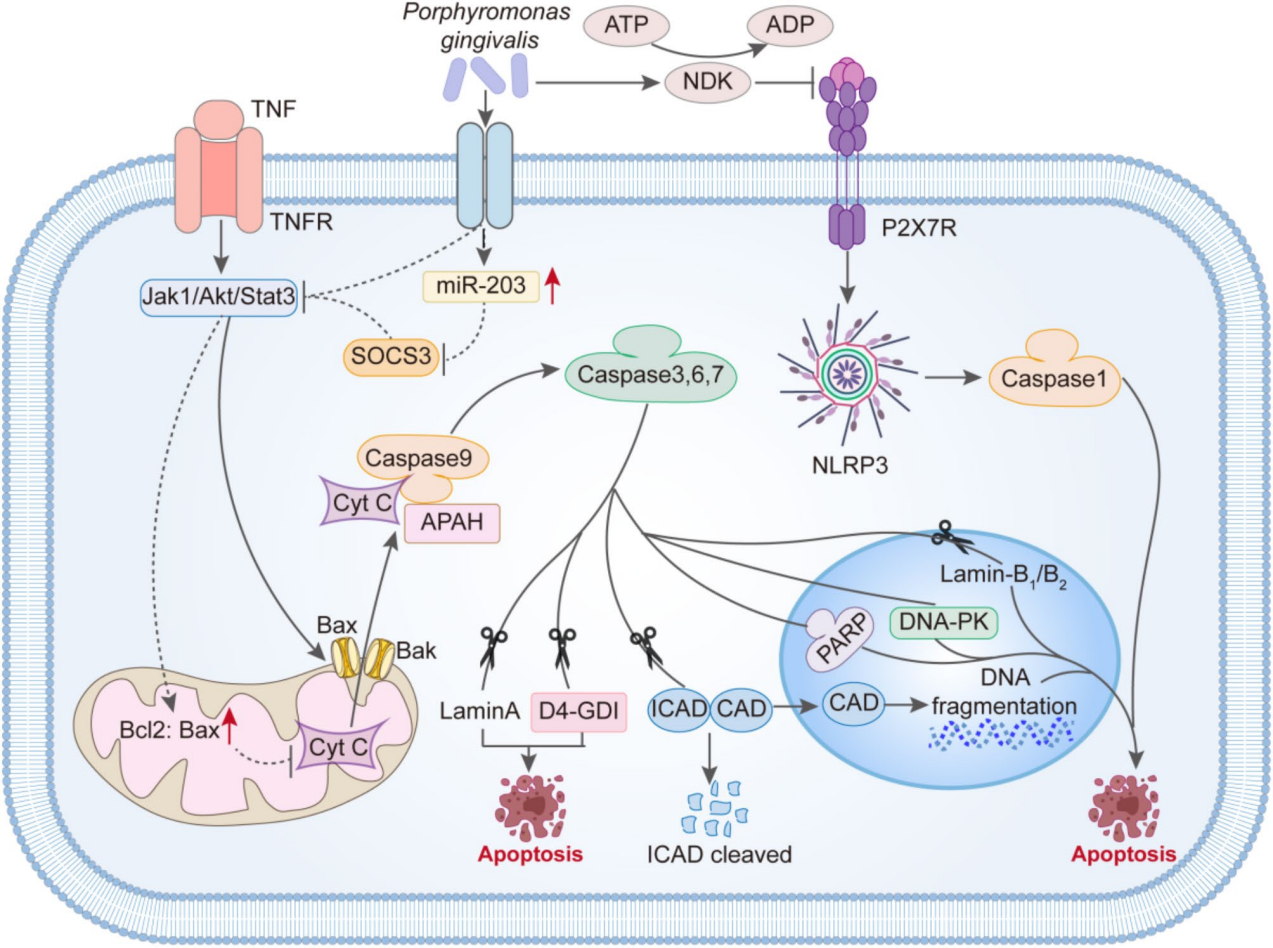
The mechanism by which Porphyromonas gingivalis inhibits apoptosis. *P. gingivalis* interferes with the signaling of the Jak 1/Akt/Stat 3 pathway, suppressing the activity of the pro-apoptotic protein Bad on the mitochondrial membrane and increasing the ratio of Bcl-2 (anti-apoptotic) to Bax (pro-apoptotic). As a result, there are abnormal mitochondrial membrane permeabilities, leading to a reduced release of Cyt C, the “core” of mitochondrial-mediated apoptosis, which ultimately blocks the activation of downstream Caspase-9 and the executor of apoptosis, Caspase-3. Furthermore, *P.gingivalis* can upregulate the expression of miR-203, thereby inhibiting the important factor SOCS3 in the Jak 1/Akt/Stat 3 pathway, contributing to the suppression of apoptosis. Lastly, *P.gingivalis* can secrete nucleoside diphosphate kinase (NDK) to prevent apoptosis mediated by the purinergic receptor P2 × 7

For example, *P. gingivalis* exhibits strong anti-apoptotic effects and can even inhibit chemically induced cell apoptosis(Fig. 2). It is well known that mitochondria-mediated cell apoptosis is a critical part of the apoptotic pathway. After apoptosis stimulation, the Bax/Bak complex inserts into the outer mitochondrial membrane pore, leading to changes in mitochondrial membrane permeability. Then, Cytochrome C (Cyt C) is released from the mitochondria into the cytoplasm and combines with apoptotic protease-activating factor 1 (Apaf-1) to form the apoptosome. The apoptosome then activates the Caspase-9 precursor, subsequently activating Caspase-3 and Caspase-7, triggering a Caspase cascade reaction, and ultimately inducing cell apoptosis. In this process, the Jak1/Akt/Stat3 pathway is one of the important pathways controlling the mitochondrial apoptotic pathway. And *P.gingivalis* interferes with the signal transduction of the Jak1/Akt/Stat3 pathway, inhibiting the pro-apoptotic activity of Bad on the mitochondrial membrane and increasing the ratio of Bcl-2 (anti-apoptotic) to Bax (pro-apoptotic). This results in abnormal mitochondrial membrane permeability, and then, the release of the “core” of mitochondrial-mediated cell apoptosis—Cyt C is reduced. Naturally, the blockade of downstream activation of Caspase-9 and the executor of apoptosis, Caspase-3, occurs. Furthermore, *P.gingivalis* can upregulate the expression of miR-203, thereby inhibiting the important factor SOCS3 in the Jak1/Akt/Stat3 pathway, ultimately participating in the inhibition of apoptosis. Additionally, in various apoptotic processes, the need for ATP is unavoidable. Interestingly, Yilmaz and others found that *P.gingivalis* can secrete nucleoside diphosphate kinase (NDK), which acts as an ATPase to degrade ATP and can also prevent apoptosis mediated by the purinergic receptor P2 × 7.

### Chronic inflammation

Inflammation, a protective host defense mechanism against external stimuli, exists in acute and chronic forms. While acute inflammation serves as the body’s initial defense, promoting pathogen elimination and tissue repair, its persistence can transition into chronic inflammation, a well-established driver of tumorigenesis and progression. Chronic inflammation exerts dual oncogenic effects: (1) through immunosuppression via T-cell inhibition and TAM polarization mediated by the STAT3 pathway, and (2) via cytokine release, such as IL-1β, IL-6, IL-12, and TNF-α, that activates oncogenes (Denk and Greten 2022), induces DNA damage, generates ROS, and dysregulates key signaling pathways including NF-κB, Kras, and P53 (particularly Kras). Furthermore, it triggers epigenetic modifications, such as DNA methylation and histone modification, that contribute to cancer development, invasion, metastasis, and drug resistance, establishing various cancers, including PDAC, as inflammation-driven diseases.

The TLR, NLR, and RIG-I-like receptor (RLR) families are the three main sensor families responsible for detecting foreign pathogens. TLRs and NLRs primarily detect bacterial and fungal PAMPs (e.g., LPS, LTA), while RLRs specialize in viral detection. For example, *F. nucleatum* is considered a potent pro-inflammatory bacterium. By acting on TLRs, it activates downstream signaling pathways, mainly NF-κB, promoting the release of cytokines such as IL-1β, IL-6, and TNF. These cytokines establish a positive feedback loop through PI3K-AKT signaling, creating a self-perpetuating inflammatory cycle (Caronni et al. 2023; Takeuchi et al. 2013). Additionally, certain cytokines like PGE2 and IL-1β interact closely with TAMs in the tumor microenvironment, positioning the PGE2—IL-1β axis as a promising therapeutic target for PDAC (Whitmore and Lamont 2014).

Another “star” in the oral-gut microbiota axis, *P. gingivalis*, exhibits more subtle behavior in inflammation, displaying both pro-inflammatory and anti-inflammatory effects (Ogrendik 2023). It can promote chronic inflammation through B7-H1 and B7-DC receptor induction, while simultaneously suppressing early inflammation via SerB-mediated dephosphorylation of NF-κB RelA/p65, which inhibits IL-8 transcription. This paradoxical suppression may facilitate microbial aggregation and subsequent severe inflammation (Jamasbi et al. 2022). Nevertheless, *P. gingivalis* has a significant disruptive effect on local host defense mechanisms (Ogrendik 2023). Moreover, the arginine and lysine-specific proteinases produced by *P. gingivalis* can convert L-arginine to L-citrulline. Citrulline has been shown to promote the release of inflammatory cytokines by altering the three-dimensional structure and function of proteins (Wang et al. 2023).

While we study how intratumoral microbes promote chronic inflammation by examining the mechanisms of specific pathogens, it is essential to note that intratumoral microbes do not act alone. Instead, they work together as a community, collaboratively promoting inflammation and maintaining the stability of the microbial population. This collaborative nature underscores the complexity of microbial contributions to tumor-associated inflammation.

### Cell cycle

Uncontrolled cell division, a hallmark of cancer, stems from cell cycle dysregulation. The precisely regulated cell cycle ensures faithful DNA replication and transmission, with any disruption potentially causing genetic defects. Emerging evidence demonstrates microbial interference in cell cycle progression as a tumorigenic mechanism (Yuzhalin 2019). In a study of gingival epithelial cells, it was found that FimA adhesin of *P. gingivalis* directly modulates cyclin-CDK complex activity, altering cell cycle phases. Specifically, *P. gingivalis* increases S-phase populations in gingival epithelial cells, suggesting G1/S transition manipulation (Wang et al. 2023). Furthermore, *P. gingivalis* can alter the phosphorylation of the cell cycle checkpoint protein p53 via LPS, leading to reduction in the activity of p53. A decrease in activity of p53 allows erroneous DNA to proceed to the next stage of the cell cycle, and as these errors accumulate over multiple cycles, the likelihood of cancer occurrence increases (Arthur et al. 2014). In addition, regarding p53, Yuzhalin AE et al. found that the arginine protease 4 (Rgp4) from the “red complex,” which includes *P. gingivalis*, *Tannerella forsythia*, and *Treponema denticola*, disrupts p53-ING4 binding, suppresses p53 acetylation, and consequently inhibits downstream p21 expression and function (Dejea et al. 2018).

DNA damage is common in cancer development, and some studies have found that intratumoral microbes’ exotoxins and metabolites play a significant role in DNA damage. Two gut-derived bacteria—B2-type *Escherichia coli* and *Bacteroides fragilis* have been reported to be involved in the process of damaging DNA. Arthur JC et al. found that the colibactin toxin (also referred to as chemical dark matter) secreted by B2-type *Escherichia coli may* cause breaks of DNA double-strand and chromosomal aberrations. Similarly, *Bacteroides fragilis* secretes Bacteroides fragilis toxin (BFT), not only directly cause DNA damage but also increase ROS levels (Luo et al. 2020; Kumavath et al. 2024). ROS exacerbates DNA damage via oxidative stress while activating PI3K/Akt signaling to promote tumor cell proliferation and survival (Channon et al. 2022). Additionally, Kumavath et al. have noted that the cytotoxic distending toxin (CDT) secreted by *Proteus* contributes to tumorigenesis by inducing DNA breaks and hyperploid (Domenis et al. 2019). Oral-derived microbes, such as *P. gingivalis* and *Tannerella forsythia*, possess PADs (peptidyl arginine deiminases), as previously mentioned, which can cause mutations in the arginine residues of oncogenes like Kras and tumor suppressor genes like p53, ultimately leading to PDAC.

### Tumor cell invasion and metastasis

M. Frydrychowicz and his colleagues discovered that intratumoral microbes can influence PDAC metastasis. Small extracellular vesicles (sEVs) are phospholipid bilayer-structured vesicles secreted by PDAC cells. These vesicles carry DNA, RNA, lipids, and proteins, participating in extracellular matrix remodeling and angiogenesis. They also mediate communication between PDAC cells and immune cells in the surrounding microenvironment to establish an immunosuppressive environment (Murota and Jobin 2021). Therefore, sEVs are crucial for the proliferation and metastasis of PDAC. Some intratumoral microbes (e.g., *F. nucleatum*) can upregulate sEV expression and promote pancreatic tumor metastasis by delivering proteins and miRNAs to cells and acting on TLR4 (Niland et al. 2021).

At present, the disruption of the intestinal vascular barrier by microbes has been observed in colorectal cancer (CRC) (Inaba et al. 2014). The microbiome of CRC, which includes species such as *Helicobacter pylori* and *Escherichia coli*, mostly originates from the gut and can naturally migrate to pancreatic tumor tissues. Whether these bacterial species can promote tumor metastasis in PDAC by breaking down the vascular barrier, as they do in CRC, remains an unsolved question.

Matrix metalloproteinases (MMPs) have long been a central topic in cancer invasion. MMPs can degrade various protein components in the extracellular matrix (ECM) and, together with tissue inhibitors of metalloproteinases (TIMPs), regulate ECM homeostasis. Under normal circumstances, the ECM serves as a “barrier” that limits tumor cell invasion. However, when MMPs become dysregulated, they excessively break down the tissue barriers that defend against tumor cell invasion. This makes MMPs a “drill” that plays a critical role in tumor invasion (Uitto et al. 2005). In recent years, intratumoral microbes have been shown to regulate MMP expression during tumor development, thus closely linking them to tumor invasiveness.

*P. gingivalis* enhances the signaling of ERK1/2-Ets1, p38/HSP27, and PAR2/NF-κB pathways, significantly increasing the level of the MMP-9 precursor (pro-MMP-9). Then, *P. gingivalis* secretes gingipain and cysteine proteases that act on the PAR2 receptor, cleaving pro-MMP-9 into its active form (Chang et al. 2015). Additionally, *P. gingivalis* induces IL-8 expression, thereby upregulating MMP-1 and MMP-10. Similarly, *F. nucleatum* activates the P38 MAPK pathway, increasing the activity of MMPs (particularly MMP-9 and MMP-13) (Kholia et al. 2015; Rubinstein et al. 2013).

In research on PADs, Kholia et al. found that PAD2/4 expression is closely linked to the level of actin (Liu et al. 2022). Actin, a key cytoskeletal component, regulates cell proliferation, shape changes, and material transport. Tumor cells can exhibit higher actin levels and altered subtype composition, then promoting invasion. Therefore, the PADs brought by intratumoral microbes may contribute to abnormally elevated actin levels, facilitating tumor cell invasion.

### Other carcinogenic pathways

In addition to the pathways mentioned above, intratumoral microbes can also promote tumorigenesis through other mechanisms. The FadA adhesin of *F. nucleatum* activates the Wnt/β-catenin pathway with the help of E-cadherin (Parida et al. 2021). Wnt/β-catenin is a canonical signaling pathway that regulates proliferation, differentiation, and self-renewal of stem cells. In pancreas, it promotes the transcription of the proto-oncogene c-Myc (Yu et al. 2017). Similarly, *Bacteroides fragilis* secretes Bacteroides fragilis toxin (BFT), which cleaves E-cadherin and then activates β-catenin-mediated signaling, thereby can also promote c-Myc transcription (Binder Gallimidi et al. 2015). The overexpression of proto-oncogenes due to these factors can result in the activation of oncogenes, promoting tumorigenesis.

In recent years, the role of microRNAs (miRNAs) in cancer has gained significant attention. *F. nucleatum* activates NF-κB and MyD88 pathways through TLR4, acting on RASA1 and increasing the expression of miR-4802 and miR-18a, which have been shown to be involved in tumorigenesis (Wang et al. 2014a, b, c). Additionally, in a new oral cancer mouse model, *P. gingivalis* promotes IL-6 expression by activating JAK2 and GSK3-β pathways, which in turn activates STAT3 phosphorylation. This feedback loop acts as an effector for stimulating oral squamous cell carcinoma (OSCC), enhancing tumor growth and invasion (Yee et al. 2014; Wang et al. 2014a, b, c; Sanchez-Vega et al. 2018; Scarà et al. 2015). However, the role of this pathway in PDAC remains to be determined.

With the development of pan-cancer signaling pathway analysis techniques, the Pan-Cancer Atlas, published in *Cell*, has revealed more and more pathways involved in tumorigenesis (Avanzini et al. 2020). Future research into the connection between these pathways and intratumoral microbes could open new doors toward the “dream of curing cancer.”

In summary, the relationship between intratumoral microbes and the development of PDAC is extremely complex. This review has only scratched the surface, and many more fascinating and surprising mechanisms are hidden beneath the iceberg, waiting to be uncovered and explored.

## The role of intratumoral microbiota in driving drug resistance in PDAC

Therapeutic resistance remains a significant barrier to effective cancer treatment, driven by complex mechanisms that include tumor-intrinsic factors and the dynamic interplay between cancer cells and their microenvironment. Among these, the intratumoral microbiota has emerged as a critical contributor to both chemoresistance and immunoresistance, shaping the therapeutic landscape of PDAC through multifaceted interactions.

In chemotherapy, microbial enzymatic activity directly inactivates drugs such as gemcitabine, a first-line chemotherapeutic agent. Specifically, gamma-proteobacteria in PDAC express cytidine deaminase (CDD), which metabolizes gemcitabine into its inactive form (2’, 2’-difluorodeoxycytidine), thereby reducing drug efficacy and promoting chemoresistance. Beyond direct drug modification, intratumoral microbes enhance chemoresistance via biofilm formation and induction of drug efflux pumps.

In immunotherapy, the role of intratumoral microbiota is equally profound, mediated through immune checkpoint modulation and the remodeling of immunosuppressive tumor microenvironment. For example, *F. nucleatum* enhances immune evasion by inducing PD-L1 overexpression on tumor cells via IFIT1-related signaling pathways, thereby conferring resistance to anti-PD-1 therapy (Gao et al. 2023).

These findings underscore the pivotal role of intratumoral microbiota in driving therapeutic resistance, highlighting their capacity to modulate both chemotherapeutic and immunotherapeutic outcomes. Given their profound impact, targeting the intratumoral microbiota represents a promising strategy to overcome resistance and enhance the efficacy of existing cancer therapies, potentially transforming the paradigm of PDAC treatment.

## Diagnosis of PDAC and the oral-gut microbiome axis: the hypothesis of a microbial pre-cancerous state

The primary challenge in tackling PDAC lies in its early, non-specific symptoms. Even with regular health check-ups, most people do not undergo ultrasound or biopsy for the pancreas without symptoms. By the time PDAC is detected, it is often in its advanced stages, which is why it remains known as the “king of cancers.” Therefore, developing simple and implementable diagnostic methods for early screening is critical to improving treatment outcomes and survival rates for PDAC patients. The traditional marker, carbohydrate antigen 19 − 9 (CA19-9), used for diagnosing and treating PDAC, is known for its low sensitivity and specificity (Farrell et al. 2012). Researchers are thus exploring better diagnostic alternatives.

In recent years, the concept of “numerical superiority” has gained attention in cancer diagnosis, particularly concerning low-biomass components. As part of the tumor, the low-biomass intratumoral microbiome has shown great potential for diagnosing PDAC. Advances in 16 S rRNA gene sequencing have revealed significant differences in the microbial abundance between healthy and pancreatic tumor tissues. A comparative study of salivary microbiomes (*n* = 10) identified Neisseria elongata and Streptococcus mitis as significantly differential species between PDAC patients and healthy controls. When combined as diagnostic markers, these bacteria demonstrated 82.1% specificity and 96.4% sensitivity, underscoring their potential for PDAC detection (Pushalkar et al. 2018).

More 16 S rRNA gene sequencing studies further identified that the bacterial phyla *Proteobacteria*, *Bacteroidetes*, *Firmicutes*, and the families *Enterobacteriaceae*, *Pseudomonadaceae*, *Streptomycetaceae*, *Myxococcaceae*, and *Clostridiaceae* were significantly more abundant in pancreatic tumor tissues compared to normal tissues, while other bacterial abundances showed no statistical significance (Riquelme et al. 2019; Yang et al. 2021; Wei et al. 2020). Fungal analysis showed increased abundance of Ascomycota and Basidiomycota phyla in tumors. Quantitative analysis demonstrated approximately 1000-fold bacterial and 3000-fold fungal expansion in PDAC versus healthy tissues. Notably, Proteobacteria, constituting only 8% of gut microbiota in PDAC patients, represented nearly 50% of intratumoral microbiota, while Malassezia fungi showed preferential tumor localization. These distinct microbial signatures highlight their potential as diagnostic biomarkers. Since the 1990s, endoscopic ultrasound-guided fine-needle aspiration (EUS-FNA) has emerged as the primary diagnostic tool for PDAC in non-surgical candidates and has facilitated intratumoral microbiota research (Fan et al. 2018). However, it is important to recognize that both EUS-FNA and surgical biopsies remain invasive procedures primarily limited to diagnostic and research applications, with minimal impact on early cancer screening.

One key characteristic of early screening is convenience, making the ease of sampling a crucial factor. The oral-gut microbiome axis, serving as the primary source of pancreatic microbial communities, offers a non-invasive sampling approach for early PDAC screening. This bidirectional interaction facilitates microbial translocation between the pancreas and the oral-gut regions through anatomical structures and/or the bloodstream. When cancer develops, changes in the pancreatic microbiome are likely to manifest throughout this axis, with saliva and fecal samples representing the most accessible sampling points. For saliva microbiomes, A.L. Wei et al. discovered that PDAC patients had significantly elevated levels of *Porphyromonas*, *Fusobacterium*, *Streptococcus*, and *Leptotrichia* compared to healthy controls, while *Neisseria* and *Veillonella* levels were reduced (Sun et al. 2020). A larger study (361 patients, 371 controls) identified *Aggregatibacter* and *P. gingivalis* as risk correlates, while *Neisseria mucosa* and *Fusobacterium periodonticum* showed positive associations with cancer likelihood (Riquelme et al. 2019). As for fecal microbiomes, the phyla *Actinobacteria*, *Proteobacteria*, and *Firmicutes*, along with their metabolic products, were found to be significantly reduced, potentially serving as the basis for early PDAC screening (Huang et al. 2022). Based on these findings, in the future, we may be likely to propose a new concept: “the microbiota-associated precancerous condition”. Analogous to the definition of “precancerous condition,” this concept refers to a certain level of microbial enrichment (such as the overabundance of *Malassezia* in PDAC) that correlates with an increased risk of malignancy, in order to offer a new approach to PDAC diagnosis and management.

Additionally, as an extension of liquid biopsy, detecting microbial plasma antibodies is gaining attention. Initially, traditional liquid biopsies focused on tumor-related DNA, RNA, and proteins shed by the tumor, with less emphasis on intratumoral microbiota. However, as more studies have highlighted the relationship between oral microbial antibodies and PDAC risk, researchers have started to recognize the potential of microbial markers in the blood as valuable resources for diagnosis and prognosis (Kohi et al. 2022). Some researchers also suggest that microbial-related metabolites, such as propionate, butyrate, PYY, and Bcl3, could be useful for PDAC diagnosis and classification, although the cancer-specific nature of these changes has yet to be fully elucidated.

It is important to note that while there has been significant progress in this field, and more researchers are exploring the feasibility of using salivary and gut microbiomes as early diagnostic markers for PDAC, there are still challenges. One major issue is sampling bias due to the low-biomass nature of microbial components and the convenience of selecting the beginning and end points of the digestive tract for sampling. Specifically, salivary and fecal microbiomes interact more with external environments, potentially causing microbial shifts due to non-cancer-related factors. For example, Feng et al. demonstrated that gut microbiota remains highly stable, even after aggressive interventions like enemas. However, oral microbiota are frequently influenced by everyday activities such as rinsing, drinking, eating, brushing teeth, and environmental factors like temperature and humidity. Similar studies in healthy populations have shown that oral microbiota experience large changes after intensive stimuli like dental cleaning but return to pre-cleaning state within a day. In conclusion, ensuring that salivary and fecal microbiomes accurately reflect PDAC-related changes is still a significant challenge.

## Prognosis of PDAC and the oral-gut microbiome axis

Current PDAC prognosis relies primarily on pathological biopsy from EUS-FNA or surgical samples. However, emerging evidence highlights the prognostic value of intratumoral microbiota α-diversity. Comparative 16 S rRNA sequencing studies reveal that long-term survivors (LTS, > 5 years) exhibit greater α-diversity than short-term survivors (STS). LTS patients show increased abundance of *Clostridium*, *Mycobacterium*, *Streptomyces*, and *Pseudoxanthomonas*, while STS patients demonstrate higher levels of *Fusobacterium* and *Bacteroides* (Geller et al. 2017). This α-diversity-prognosis correlation was consistently observed in both Western and Chinese PDAC cohorts, despite slight variations in dominant bacterial species, potentially attributable to genetic, racial, dietary, and geographical factors (Lehouritis et al. 2015).

Based on these studies, Riquelme et al. developed a prognostic model using *Clostridium*, *Mycobacterium*, *Streptomyces*, and *Pseudoxanthomonas*, which effectively predicted the prognosis of PDAC patients in cohorts from the Anderson Cancer Center (AUC = 97.51) and Johns Hopkins Hospital (Geller et al. 2017). However, the prognostic implications of α-diversity vary across cancer types, as evidenced by its association with poor outcomes in gastric adenocarcinoma. Therefore, when developing microbial-based prognostic models for PDAC, comprehensive consideration of race, genetics, customs, geography, and cancer subtype is essential.

## The potential of the oral-gut microbiome axis in PDAC treatment

Despite surgical intervention being the primary curative approach for PDAC, its application is often limited by late-stage diagnosis, tumor unresectability, and high postoperative recurrence rates. Consequently, chemotherapy and immunotherapy remain the cornerstone of clinical management. However, their therapeutic efficacy is frequently constrained by incomplete tumor suppression, metastatic progression, and significant treatment-related toxicities. These limitations have catalyzed the exploration of alternative strategies, with the intratumoral microbiota emerging as a promising therapeutic frontier. Recent advances in microbiome research have elucidated its multifaceted role in PDAC pathogenesis, including tumor microenvironment (TME) modulation, chronic inflammation induction, cellular proliferation dysregulation, and metastasis promotion. Furthermore, emerging evidence demonstrates the microbiota’s capacity to influence both chemotherapeutic and immunotherapeutic responses, positioning it as a novel therapeutic target in PDAC management.

### Targeting the oral-gut microbiome axis to boost PDAC therapy

Immunotherapy revolutionizes cancer treatment by harnessing the immune system to target and destroy tumor cells. In PDAC, it seeks to counteract the immunosuppressive tumor microenvironment and amplify antitumor immunity. Key approaches include immune checkpoint inhibitors (ICIs) targeting PD-1/PD-L1 and CTLA-4 pathways to reactivate cytotoxic T cells, alongside emerging adoptive cell therapies (e.g., CAR T cells) and cancer vaccines for tumor-specific immunity. However, therapeutic efficacy remains limited due to dense stromal barrier, low tumor mutational burden, and abundant immunosuppressive cells (Tregs, TAMs). Recent advances highlight the oral-gut microbiome axis as a pivotal modulator of immune responses, providing novel opportunities to optimize immunotherapy outcomes in PDAC. The oral-gut microbiome axis influences systemic and local immunity, thereby impacting the outcomes of immunotherapy. Specifically, intratumoral microbiota can reshape the TME by promoting immune cell infiltration and activation, while also modulating the expression of immune checkpoints. Similarly, the gut and oral microbiomes play a crucial role in regulating systemic immune homeostasis, with specific microbial communities enhancing or suppressing antitumor immunity.

Chemotherapy, another “weapon” of cancer treatment, employs cytotoxic agents to eliminate rapidly dividing tumor cells and suppress malignant proliferation. In PDAC, chemotherapy serves as first-line therapy for both early and advanced cases, utilizing agents like gemcitabine and FOLFIRINOX. However, its efficacy is significantly limited by the tumor’s dense stromal matrix that impedes drug penetration, inherent/acquired chemoresistance mechanisms, and systemic toxicity affecting healthy tissues. These challenges contribute to suboptimal treatment responses and poor prognosis in most patients. Emerging evidence highlights the oral-gut microbiome axis may be a critical modulator of chemotherapeutic outcomes through regulation of drug activation/inactivation and impact on drug delivery.

Therefore, targeting these microbial populations—whether through direct modulation of intratumoral microbiota or interventions aimed at the oral and gut microbiomes—holds promise for overcoming therapeutic resistance.

#### Targeting intratumoral microbiota

The ablation of microorganisms in tumors mediated by specific antibiotics reshapes the immune microenvironment and enhances the immune function to kill and eliminate tumor cells. Beyond direct effects, microbial ablation will also reduce the impact of microorganisms on the effectiveness of chemotherapy and ultimately inhibit the progression of PDAC (Kostic et al. 2013). However, it is worth noting that not all intratumoral microorganisms have a negative impact on chemotherapy. P. Lehouritis et al. found that another intratumoral microorganism, *Escherichia coli*, can actually reduce the toxic and side effects of some chemotherapy drugs (such as doxorubicin, gemcitabine, and arabinosine) on patients (Nalluri et al. 2021).

In terms of immunotherapy, three microorganisms closely associated with long-term survival in patients—*Pseudoxanthomonas*, *Streptomyces*, and *Mycobacterium*—were found to promote antitumor immunity by recruiting and activating CD8 + T cells and polarizing CD4 + T cells towards the Th1 phenotype (Kostic et al. 2013). Additionally, they upregulated PD-1 expression, increasing the responsiveness of PDAC cells to chemotherapy. Similarly, *Bifidobacterium* was shown to accumulate in tumors via interferon-dependent and STING (stimulator of interferon genes) mechanisms, enhancing anti-CD47 immunotherapy (Gough et al. 2011). These findings suggest the potential of targeting intratumoral microbiota in PDAC treatment. However, it is noteworthy that samples from patients treated with neoadjuvant chemotherapy exhibited significantly elevated levels of *Enterobacteriaceae* compared to those treated with gemcitabine alone or without prior chemotherapy. This observation implies that chemotherapy may intrinsically reshape the intratumoral microbiota composition in PDAC patients, highlighting a potential interplay that necessitates further investigation (Genton et al. 2021).

#### Targeting gut and oral microbiota

The oral-gut microbiome axis and the intratumoral microbiome in PDAC are interconnected, with changes in one potentially influencing the other. Given the relative ease of obtaining samples from these two sites, microbiota interventions in these areas are also feasible. Fecal microbiota transplantation (FMT), for instance, has a long history in disease treatment. Over 2,000 years ago, Chinese practitioners used an oral “yellow soup” derived from healthy individuals’ fecal slurry to treat severe diarrhea, and this practice was later adopted in Africa during World War II (Mohindroo et al. 2021). Although FMT use in cancer patients is currently limited, two clinical trials involving melanoma patients who failed immunotherapy have shown that FMT can positively affect the disease by modulating gut microbiota. Moreover, Riquelme et al. found that FMT from LTS of PDAC enhanced immune responses in mouse models, whereas FMT from STS did not yield similar effects (Weniger et al. 2021).

In contrast, interventions targeting oral microbiota are less explored, despite their significance as a source of intratumoral microbiota in PDAC. Maintaining oral hygiene and microbial homeostasis has been linked to improved prognoses in systemic diseases like diabetes and lupus (Khor et al. 2021). However, achieving long-term stability in oral microbiota is challenging. Systemic antibiotics often fail to effectively target oral microbiota, while topical treatments can impair patient quality of life and adherence. The development of new materials offers potential solutions. For example, enzyme/temperature/light-responsive hydrogels for dental restorations can release bioactive agents, such as antibiotics, under specific conditions to regulate the oral microenvironment and sustain long-term health. Similarly, novel bioceramic materials have shown efficacy in inhibiting *P. gingivalis*, a bacterium strongly associated with PDAC. Thus, for PDAC patients, particularly those with oral conditions like cavities, such materials could help reduce harmful oral microbes implicated in cancer progression.

Generally speaking, while the effects of the above strategies on altering the intratumoral microbiome remain to be further explored, they may play a substantial role when used in combination with targeting intratumoral microbiota in future PDAC treatments. Here, we summarize clinical trials of the microbiome in pancreatic cancer(Table 2).

**Table 2:** The clinical trials of the Microbiome in pancreatic cancer

NCT Number	Study Title	Study Status	Conditions	Interventions	Phases
NCT04274972	The Microbiome of Pancreatic Cancer: “PANDEMIC” Study	RECRUITING	Microbial Colonization|Pancreas Cancer|Pancreas Infection|Pancreas; Fistula	DIAGNOSTIC_TEST: Microbiome evaluation	–
NCT03302637	Oral Microbiome and Pancreatic Cancer	COMPLETED	Pancreatic Cancer	OTHER: 16 S rRNA gene sequencing assay	–
NCT04922515	Pancreatic Ductal Adenocarcinoma - Microbiome as Predictor of Subtypes	UNKNOWN	Pancreatic Cancer	OTHER: Oral and rectal swabs for microbiome sequencing	NA
NCT05462496	Modulation of the Gut Microbiome With Pembrolizumab Following Chemotherapy in Resectable Pancreatic Cancer	RECRUITING	Pancreatic Cancer	PROCEDURE: Biopsy|DRUG: FOLFIRINOX|DRUG: Ciprofloxacin|DRUG: Metronidazole|DRUG: Pembrolizumab|PROCEDURE: Surgical Resection	PHASE2
NCT04993846	Pancreatic Cancer and Oral Microbiome	UNKNOWN	Oral Microbiome|Pancreatic Cancer|IPMN|Parodontopathy	DIAGNOSTIC_TEST: Dental plaque sampling|DIAGNOSTIC_TEST: qPCR	–
NCT05462314	Intestinal Microbiome, Oral Microbiome, and Whole Blood Transcriptome Analyses in Gastrointestinal Malignancies	RECRUITING	Gastrointestinal Cancer|Pancreatic Cancer|Esophageal Cancer|Gastric Cancer|Rectal Cancer|Liver Cancer|Biliary Cancer		–
NCT06381882	The Role of the Human Microbiome in Patients After Pancreatic Resection.	NOT_YET_RECRUITING	Pancreas Cancer|Pancreas Neoplasm|Pancreas Adenocarcinoma|Periampullary Cancer|Periampullary Carcinoma|Microbial Colonization	PROCEDURE: Pancreatic resection	–
NCT06319755	Characteristics of Intestinal Microbiome Following Pancreatic Surgery	NOT_YET_RECRUITING	Pancreatic Cancer|Microbiota|Pancreatoduodenectomy	–	–
NCT03891979	Gut Microbiome Modulation to Enable Efficacy of Checkpoint-based Immunotherapy in Pancreatic Adenocarcinoma	WITHDRAWN	Pancreatic Cancer	DRUG: Pembrolizumab|DRUG: Ciprofloxacin 500 mg PO BID days 1–29|DRUG: Metronidazole 500 mg PO TID days 1–29	PHASE4

### Specific approaches to enhance therapeutic efficacy

#### Antibiotics

As the backbone of bacterial growth inhibition, antibiotics have long been considered by researchers. Antibiotics can suppress PDAC progression by disrupting harmful tumor microbiota, thereby improving patient outcomes. Preclinical studies in PDAC mouse models revealed that the administration of antibiotics to deplete gut and tumor microbiota was found to affect PDAC progression (Knight et al. 2023). Clinically, a retrospective study of 580 patients found that advanced PDAC patients who used antibiotics for more than 48 h had longer overall survival (OS) and progression-free survival (PFS) compared to non-users (Corty et al. 2020).

The concept of combination therapy is also gaining traction, with antibiotics being combined with chemotherapy drugs. Macrolide antibiotics can reduce resistance to gemcitabine in PDAC caused by *Klebsiella pneumoniae*, and quinolone antibiotics have shown similar effects (Seelbinder et al. 2020). A retrospective study of 430 PDAC patients also found that those treated with antibiotics in combination with gemcitabine had better outcomes than those treated with gemcitabine alone. These results underscore the potential of antibiotic-chemotherapy synergism in addressing microbial-mediated resistance mechanisms and optimizing therapeutic efficacy in PDAC. Further research is warranted to elucidate the underlying mechanisms and refine combination strategies for broader clinical application.

Despite the promising theoretical and experimental results, the side effects of antibiotics cannot be ignored. Several studies have pointed out that combining quinolone antibiotics with gemcitabine increases the risk of gastrointestinal side effects, obesity, and liver function abnormalities, while β-lactam antibiotics increase the risk of hematologic adverse events (Hajishengallis and Lamont 2012). Additionally, long-term use of antibiotics can disrupt the normal microbiome, increasing the risk of dysbiosis and opportunistic infections (Chen et al. 2020). Therefore, an ideal approach would be to target specific bacterial strains that promote PDAC while preserving strains that inhibit its progression. However, microbes often function as communities, so targeting one specific microbe may reduce its abundance temporarily, but the remaining community might restore its functional microbial ecosystem, leading to a rebound effect over time (Konishi et al. 2021). Therefore, the strategy of using antibiotics merits further research, and developing novel antibiotics may be a more feasible approach.

#### Probiotics and prebiotics

Since the FAO/WHO first defined probiotics in 2001, they have been increasingly used to treat gastrointestinal diseases. Given that microbiota from the oral-gut microbiome axis can enter the pancreas via the pancreatic duct, oral administration of probiotics offers a potential treatment strategy, although this still requires more clinical trials to validate. Chen et al. found that *Lactobacillus* reduced the number and grade of pancreatic intraepithelial neoplasia, delayed pancreatic tumor growth in mice, and inhibited the EMT process, thereby reducing metastasis (Wang et al. 2014a, b, c). Similarly, *Lactobacillus* also inhibited *P. gingivalis*-induced pancreatic tumor growth in mice (Taper and Roberfroid 2005). The metabolic product of *Aspergillus*, heptanoic acid, can activate the p38 MAPK signaling pathway, inducing apoptosis in PDAC cells (Zhao et al. 2023). Additionally, probiotics can reduce *Helicobacter pylori* colonization in tumors (Turnbaugh et al. 2006). However, probiotics, as live microorganisms, may disrupt the native microbiome ecosystem and potentially compromise antibiotic efficacy when used long-term. Taper HS et al. recommend avoiding concurrent use of probiotics and antibiotics due to potential interference and increased risk of opportunistic infections. In contrast, prebiotics, such as fructooligosaccharides (FOS), selectively promote the growth of beneficial bacterial groups and modulate short-chain fatty acid (SCFA) levels (Zhao et al. 2018). Furthermore, researchers are exploring the design of PDAC-specific prebiotics to enhance bacteria associated with long-term survival while suppressing tumor-promoting species, though this remains a distant goal.

#### Dietary therapy

In recent years, dietary interventions have emerged as a promising strategy to modulate microbiota composition and metabolic processes. Studies demonstrate that significant dietary changes can rapidly reshape gut microbial communities (O’keefe 2016).For instance, a low-fat, high-fiber diet reduces Bacteroides abundance while increasing short-chain fatty acid (SCFA) production, both of which are implicated in PDAC risk (Xue et al. 2023; Canale et al. 2021). In summary, previous studies have found that high consumption of meat, fat products, and sweets is associated with a higher risk of PDAC, whereas diets rich in vegetables, fruits, soy, and fish are protective. Currently, similar studies have begun to use a high-fiber diet to prevent CRC, but such studies on PDAC are still quite scarce (Chowdhury et al. 2019).

#### Engineered bacteria

With the continuous development of nanomaterials and bioengineering technology, researchers have begun modifying natural bacteria to endow them with specific functions, creating what is known as “engineered bacteria.” These bacteria can exert antitumor effects through various mechanisms, which can be summarized as: (1) directly inducing apoptosis or autophagy in cancer cells, (2) inducing immune responses, (3) regulating tumor metabolic processes, and (4) delivering drugs to tumor sites (Gurbatri et al. 2022). For example, Canale et al. used bioengineering techniques to modify *E. col*i *Nissle 1917* (EcN), enabling the bacteria to convert nitrogenous compounds within tumors into L-arginine, thus promoting T-cell-mediated immune responses (Cieplak et al. 2018). Similarly, Chowdhury et al. designed a non-pathogenic *E. coli* strain capable of synthesizing CD47 nanobodies, which adhere to the surface of cancer cells, reducing tumor cell immune evasion (Mondal et al. 2020). Moreover, the antitumor effects of *Salmonella typhimurium* on mouse tumors are also being gradually explored (Marelli et al. 2021). Overall, the development and application of engineered bacteria represent significant advancements in cancer treatment.

Tumor-colonizing bacteria can activate tumor immunity by secreting surface components and intracellular heterologous proteins, such as FlaB from the ΔppGpp strain, which promote M1-like macrophage activation while reducing anti-inflammatory M2 macrophage numbers. Additionally, engineered bacteria can enhance immunogenicity of “cold tumors” via cytokine or chemokine production to recruit T cells, exemplified by *Salmonella* expressing human IL-2 increasing NK and NKT cell counts in metastatic gastrointestinal cancer patients (*n* = 22). Advances in synthetic biology enable controlled bacterial immune therapy, including the use of nanobodies targeting specific receptors like CD47 or PD-L1 to bypass immune surveillance. Furthermore, the engineered EcN strain with increased L-arginine levels within the tumor microenvironment promotes antitumor T cell responses (L-arginine production from L-arginine-producing EcN). Finally, the expression of mutated tumor-derived antigens by antigen-presenting cells induces neoantigen-specific T cell activation. These mechanisms collectively demonstrate how synthetic biology can harness bacterial systems to enhance therapeutic immune responses against cancer (Nguyen et al. 2023).

#### Other emerging strategies: bacteriophages and oncolytic viruses

Bacteriophages and oncolytic viruses represent two promising therapeutic approaches in cancer treatment. Bacteriophages, viruses that specifically infect and lyse bacteria, are being investigated for their potential in microbiota modulation and tumor treatment. Preclinical studies have demonstrated their ability to selectively eliminate target bacteria while preserving non-targeted species (Shen et al. 2023), suggesting their potential as an adjunct to antibiotics in microbiota modulation for PDAC patients.

Meanwhile, oncolytic viruses exhibit direct antitumor effects through selective replication in tumor cells, exploiting defects in tumor suppressor genes, followed by cell lysis and subsequent infection of adjacent tumor cells. Beyond direct tumor destruction, these viruses enhance host antitumor immunity by inducing lymphocyte and antigen-presenting cells (APCs) infiltration at tumor sites. The release of tumor antigens during cell lysis further amplifies APC-mediated antigen presentation, potentially preventing tumor recurrence and metastasis (Bazan-Peregrino et al. 2021).Recent studies have highlighted the therapeutic potential of oncolytic viruses such as vaccinia virus, herpes simplex virus, and oncolytic adenovirus in PDAC (Holbrook et al. 2021; Jiang et al. 2023). However, their clinical application is currently restricted to immunotherapy-resistant cases, and further research is needed to determine whether they can enhance therapeutic efficacy and to explore their broader clinical potential.

## Conclusions

The oral and intestinal microbiota, functioning as an integrated entity termed the oral-gut microbiota axis, play a critical role in the initiation and progression of PDAC. Emerging evidence indicates that this axis may further contribute to therapeutic resistance in PDAC through multifaceted mechanisms. Based on these findings, the concept of a ‘microbiota-associated precancerous condition’ is proposed, offering a novel framework to improve early detection and diagnostic strategies for PDAC. Finally, targeting the oral-gut microbiota axis holds significant promise as a potential therapeutic avenue in the management of PDAC.

However, given the low biomass of the intratumoral microbiome and the unavoidable contamination during research, most studies on intratumoral microbiota are currently limited to observational research, yielding constrained and often inconclusive data. Furthermore, the sub-localization of intratumoral microbiota is a crucial issue worth exploring in the future, as it significantly impacts the formulation of treatment strategies. Factors such as geography, race, lifestyle, and researchers’ subjective judgments also influence the research process and strategy formulation. Despite these challenges, even at present, we must consider these factors from both both therapeutic and prognostic perspectives. Looking ahead, the integration of artificial intelligence (AI) and machine learning with large-scale multi-omics data analysis offers a promising avenue to address these complexities. Researchers may be able to disentangle the intricate relationships between microbial communities, host factors, and clinical outcomes, ultimately advancing the realization of precision medicine in oncology through AI-driven models. In conclusion, while the “oral-gut microbial axis” represents a promising frontier for the early diagnosis and treatment of PDAC, overcoming current limitations and harnessing emerging technologies will be essential to fully elucidate its potential and translate these insights into clinical practice.

## Data Availability

No datasets were generated or analysed during the current study.
